# Decades of land use change and its impact on air quality in Egypt’s Middle Nile Delta observed from space

**DOI:** 10.1038/s41598-025-17019-9

**Published:** 2025-08-28

**Authors:** Ahmed El-Zeiny, Alaa Nagy, Hoda Nour-Eldin

**Affiliations:** 1 Environmental Studies Department, National Authority for Remote Sensing and Space Sciences (NARSS), Cairo, Egypt; 2 Department of Zoology and Entomology, Faculty of Science (Girls Branch), Al-Azhar University, Cairo, Egypt; 3 Land Use Department, National Authority for Remote Sensing and Space Sciences (NARSS), Cairo, Egypt

**Keywords:** Air pollution, Land cover, MODIS, Sentinel-5P, Particulate matter, Gharbia, Climate sciences, Environmental sciences, Space physics

## Abstract

Air pollution has become one of the most pressing environmental challenges globally, with significant consequences for public health and ecosystems. Recently, Gharbia Governorate has undergone remarkable changes in land use/land cover (LULC), leading to a variety of environmental impacts. These transformations have raised concerns about their potential influence on air quality, making it crucial to investigate how shifts in LULC are affecting pollution levels in the region. Understanding this relationship is vital for developing sustainable land management strategies and improving air quality monitoring in the area. This study aims to employ remotely sensed data integrated with Geographic Information System (GIS) to monitor the LULC changes and evaluate their influences on the governorate’s air quality. Two Landsat 8 Operational Land Imager data, as OLI acquired in 2023, 2013, and Landsat 5 Thematic Mapper data, as TM 5 acquired in 2003, were used. A maximum likelihood classifier was used to produce a land cover map and track changes in land cover of the study area. Aqua, Terra, and Sentinel-5P were employed to measure the PM_2.5_, CO, NO_2,_ and SO_2_ concentrations. The classification results identified two dominant LULC classes: cultivated land and urban area. Between 2003 and 2023, urban areas increased by 136.2 km^2^, while cultivated lands declined by 135.94 km^2^, reflecting significant urban expansion. This land conversion was associated with a marked impact on air quality. PM_2.5_ concentrations ranged between 7.12 and 8.66 µg/m^3^ during the study period. Notably, NO_2_ levels peaked during autumn (3.13 µg/m^3^), while elevated CO and SO_2_ concentrations were observed in summer (946.79 µg/m^3^) and winter (8 µg/m^3^), respectively. The highest pollutant levels were consistently recorded in the recently expanded urban areas compared to other land use categories. These findings demonstrate that urban sprawl has a significant and direct impact on air quality, highlighting the need for integrated land-use planning and air pollution control. The generated data provides a valuable foundation for sustainable development and environmental decision-making in Gharbia Governorate.

## Introduction

Over the past few decades, land use and land cover (LULC) changes have emerged as a key driver of environmental transformation, influencing local weather patterns, urban air quality, and regional climate conditions. Among the most critical drivers of LULC change are urban sprawl, industrialization, and deforestation, all of which are deeply tied to rapid population growth and economic development. While urbanization supports socioeconomic advancement, it is increasingly associated with environmental degradation, particularly the deterioration of air quality. Uncontrolled urban expansion disrupts the balance between urban and rural areas, leading to overexploitation of land resources, increasing vehicular emissions, and contributing to atmospheric pollution^1–3^.

Accurate and timely information on LULC dynamics is essential for understanding the interactions between anthropogenic activities and the environment, especially in regions experiencing rapid change. In Egypt, numerous studies have used remote sensing and Geographic Information System (GIS) technologies to assess LULC trends^4–11^. However, fewer studies have examined the environmental consequences of these changes, particularly their impact on air quality.

In recent years, attention has grown regarding the interplay between LULC change and variations in ambient air pollutant concentrations. This is largely due to the adverse health and ecological effects associated with pollutants such as carbon monoxide (CO), sulfur dioxide (SO_2_), nitrogen dioxide (NO_2_), ozone (O_3_), and particulate matter (PM_10_ and PM_2.5_)^12–17^. Despite growing global interest in this relationship, relatively few studies in Egypt have systematically analyzed how land cover transformations correlate with satellite-derived air quality indicators over time.

Recent advances in remote sensing make it possible to integrate LULC monitoring with air pollution assessment using multi-source satellite data. Landsat imagery, with its moderate spatial resolution, has long been a primary tool for detecting land cover change. Meanwhile, atmospheric data from instruments such as the Moderate Resolution Imaging Spectroradiometer (MODIS) and the Sentinel-5 Precursor’s TROPOspheric Monitoring Instrument (TROPOMI) enable regional-scale monitoring of air pollutants over time^8,18–20^.

Gharbia Governorate, located in Egypt’s Nile Delta, has experienced significant urban sprawl and land conversion, particularly the encroachment of built-up areas onto fertile agricultural land. These rapid landscape changes raise critical questions about the region’s environmental trajectory and highlight the need for integrated land–air quality assessments. Therefore, this study aims to analyze LULC changes in Gharbia Governorate over 20 years (2003, 2013, and 2023) using Landsat imagery, and evaluate the spatiotemporal variations in atmospheric pollutants, including PM_2.5_, CO, NO_2,_ and SO_2_, derived from Aqua, Terra, and Sentinel-5P datasets. By linking LULC dynamics with air quality trends, this research contributes to a deeper understanding of how land development patterns affect environmental health, offering essential insights for sustainable planning and policymaking in Egypt.

## Materials and methods

### Study area

**Fig. 1:**
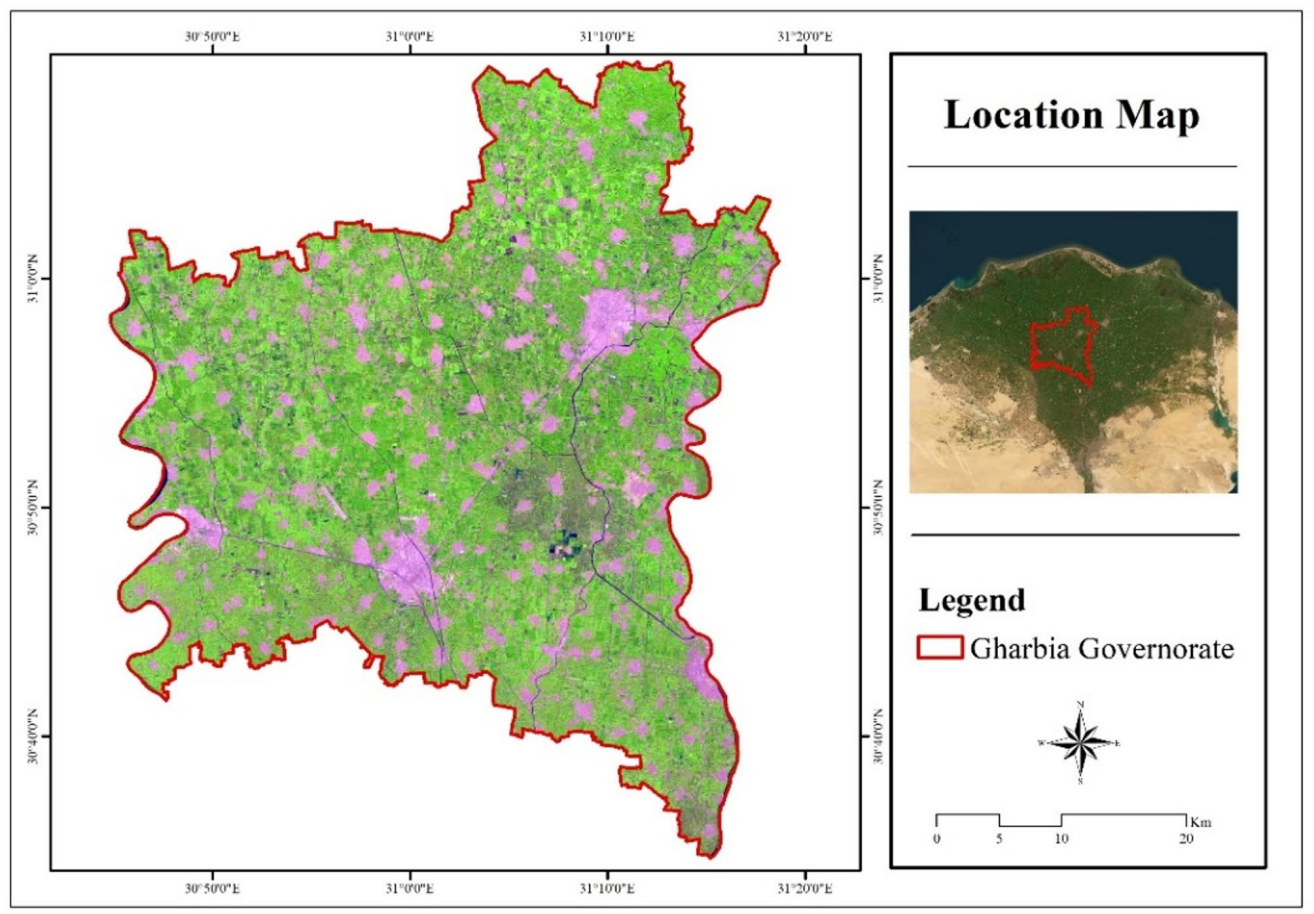
Location map of Gharbia Governorate (2023). (Generated by the authors using Landsat-8 data via ArcGIS version 10.4.1^21^.

Gharbia Governorate is located in the Nile Delta, between the Damietta and Rosetta branches, at latitudes 30° 45’ East and 30° 34’ North. The total area of Gharbia Governorate is 1943.27 km²^22^. Its population is 5.4 million, according to statistics issued by the Central Agency for Mobilization and Statistics (CAPMAS) in 2023. It consists of eight administrative districts: El-Mahala El-Kobra, Zefta, Kafr El Zayat, Alsanta, Samanoud, Kotor, and Basioun. The main irrigation canals in the governorate are Bahr Shibin, Al Bajuriyyah, Al Qasid, Al Jafariyyah, Bahr Nashart, Al Atf, As Sahil, Al Ninaiyyah, Al Rayyah Al Abbasi, and Al Rayyah Al Menoufi (Fig. 1).

### Data acquisition and preparation

#### Land use/land cover data acquisition and processing

In this study, three free downloadable images (http://glovis.usgs.gov/) were used: two Landsat 8 Operational Land Imager data (OLI) acquired on September 23, 2023, and September 11, 2013, with pixel size 30 m, and Landsat 5 Thematic Mapper data (TM) acquired on September 16, 2003 with pixel size 30 m. Table 1 shows the technical and spectral specifications of Landsat satellites. This study employed ENVI (5.3) software (L3Harris Geospatial, https://www.l3harrisgeospatial.com/Software-Technology/ENVI) to classify the images and ArcGIS (10.4.1) (Esri, https://www.esri.com/en-us/arcgis/products/arcgis-desktop/overview) to generate the land use/cover maps. The study area was covered by two scenes, 177 − 39 and 177 − 38. The images were subset using a shape file of the administrative boundary. Atmospheric correction and radiometric calibration are the basic preprocessing steps followed by correcting Landsat data. Radiometric correction must be carried out to monitor actual landscape changes as indicated by differences in surface reflectance from multi-temporal satellite imagery. Radiometric calibration refers to a set of preprocessing techniques applied to remote sensing data to ensure accurate and consistent reflectance values. These procedures are correct for factors such as sensor sensitivity, atmospheric absorption and scattering, solar angle, and topographic variations, thereby improving the reliability of subsequent analyses^23^.

Supervised classification is a technique in which pixels are categorized based on predefined training sites obtained from aerial imagery, maps, and field data^24^. In this study, the Maximum Likelihood Classifier (MLC) was employed to generate a land cover map of Gharbia Governorate, aiming to identify existing natural resources and major human activities affecting the area. MLC is one of the most widely used methods for the supervised classification of remotely sensed data due to its statistical robustness and computational efficiency^25^. It assumes that the spectral data for each class is normally distributed and utilizes both variance and covariance in the classification process, making it well-suited for moderately heterogeneous landscapes such as Gharbia Governorate.

While machine learning classifiers like Support Vector Machines (SVM) and Random Forest (RF) have demonstrated higher classification accuracy in complex landscapes^26,27^, the choice of MLC in this study was guided by its proven effectiveness with multispectral Landsat imagery, particularly when sufficient ground truth data are available^25^. To ensure classification accuracy, ground control points were used for validation. Using Global Positioning System (GPS) and original false-color composite images, specific locations in the classified maps were identified and verified through on-site inspections. The reference ground truth points were then used to assess classification accuracy. The results showed overall accuracy exceeding 90% across all years, with Kappa coefficients above 0.9, indicating a strong agreement between the classified outputs and actual land cover types. Furthermore, MLC required less computational overhead and parameter tuning, offering a more practical approach for processing multi-temporal datasets in this context.

**Table 1:** Technical and spectral specifications of Landsat satellites.

Satellite	Sensor	Bands	Wavelength (mm)	Spatial resolution (m)
Landsat-5	Thematic Mapper (TM)	Band 1 Visible Blue	0.45–0.52 μm	30
		Band 2 Visible Green	0.52–0.60 μm	
		Band 3 Visible Red	0.63–0.69 μm	
		Band 4 Near-Infrared	0.76–0.90 μm	
		Band 5 Near-Infrared	1.55–1.75 μm	
		Band 6 Thermal	10.40–12.50 μm	60
		Band 7 Mid-Infrared	2.08–2.35 μm	30
Landsat-8	Operational land Imager (OLI)	Band 1—Coastal aerosol	0.43–0.45	30
		Band 2—Blue	0.45–0.51	
		Band 3—Green	0.53–0.59	
		Band 4—Red	0.64–0.67	
		Band 5—NIR	0.85–0.88	
		Band 6—SWIR 1	1.57–1.65	
		Band 7—SWIR 2	2.11–2.29	
		Band 8—Panchromatic	0.50–0.68	15
		Band 9—Cirrus	1.36–1.38	30
		Band 10—TIRS1	10.60–11.19	100
		Band 11—TIRS2	11.50–12.51	

#### Air parameters data acquisition and processing

Aqua, Terra, and Sentinel-5P satellites were employed to measure concentrations of PM_2.5_, CO, NO_2_, and SO_2_. The MCD19A2 aerosol product was used for PM_2.5_ estimation, derived from MODIS Level-1 data with a spatial resolution of 1 km (Source: https://www.earthdata.nasa.gov/). MCD19A2 Version 6 is a daily dataset that integrates Terra and Aqua observations using the Multi-angle Implementation of Atmospheric Correction (MAIAC) algorithm (Table 2).

To analyze variations in particulate matter concentrations, Aerosol Optical Depth (AOD) values over Gharbia Governorate during winter and spring 2023 were extracted from the MCD19A2 product. The images were processed and georeferenced using the MODIS conversion toolkit in ENVI 5.3 software. PM_2.5_ concentrations were then estimated from AOD using the equation proposed by Chudnovsky et al.^28^:

$$\:{\text{P}\text{M}}_{2.5}\:=23.89\:\times\:\:\text{A}\text{O}\text{D}+5.18$$

After processing, the seasonal average, minimum, and maximum PM_2.5_ concentrations for 2023 in Gharbia Governorate were statistically analyzed.

For gas retrievals, Sentinel-5P data is freely accessible through various web services (Site: https://www.sentinel-hub.com/). The data is categorized by time into near real-time (NRT), offline (OFFL), and reprocessed (RPRO) datasets. In this study, Sentinel-5P/TROPOMI Level 2 OFFL satellite imagery from January 1 to December 31, 2023, was obtained via EO Browser (Table 2).

ArcMap software was used for data processing, with all datasets likely georeferenced to the World Geodetic System 1984 (WGS84). Each gas layer was resampled to a 1 km spatial resolution using the ArcMap toolkit, and the governorate shapefile was applied to mask the data. After processing, the seasonal average, minimum, and maximum concentrations of air pollutants (CO, NO_2_, and SO_2_) were statistically analyzed. To compare the retrieved gas concentrations with Egyptian regulatory limits, the satellite-derived values (mol/m^2^) were converted to µg/m^3^. This conversion was performed using the equation proposed by Savenets^29^ which is:

$$\:C=\:\frac{{C}_{col}}{H}\times\:M\times\:A$$

**Fig. 2:**
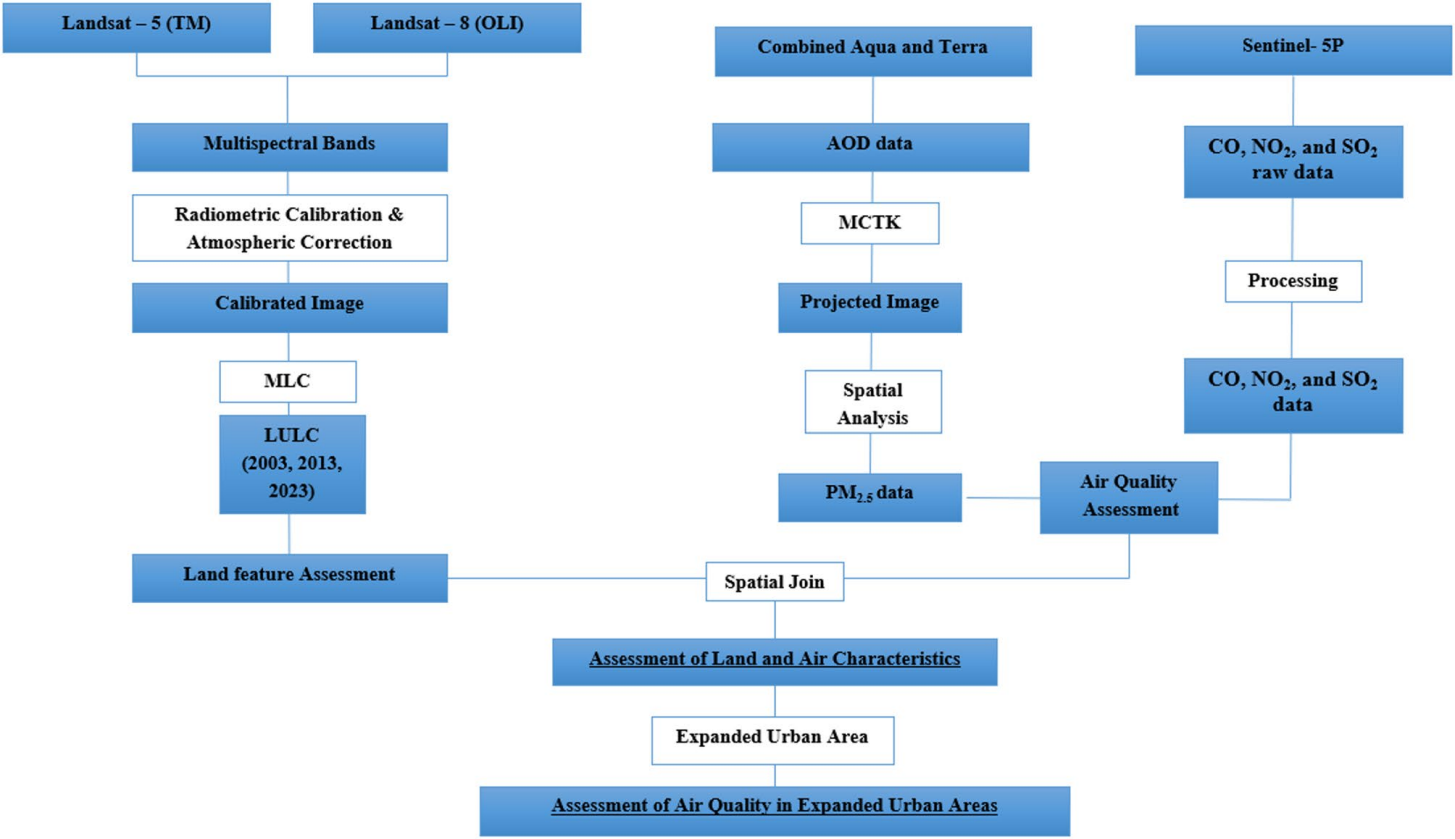
Flow chart showing methodology adopted in the study.

Where $\:{C}_{col}$ is the pollutant column content (mol/m^2^), $\:M$ is the molar mass (g/mol), $\:A$ is a constant equaling 1000, for conversion from g/m^3^ to mg/m^3^,$\:\:H$ is expressed in m. A similar method was successfully utilized by Nagy et al.^30^. in monitoring air pollution in the other Delta Governorates, Sharkia. The process adopted in this study is summarized in the flowchart shown in Fig. 2.

**Table 2:** Technical and spectral specifications of Aqua, Terra, and Sentinel-5P satellites.

Satellite	Sensor	Bands	Wavelength (µm)	Spatial resolution (m)
Combined Aqua and Terra	Moderate resolution imaging spectroradiometer (MODIS)	36	620–965 nm (Bands 1–19) 3.660–14.385 (Bands 20–36)	250 m (Bands 1–2) 500 m (Bands 3–7) 1000 m (Bands 8–36)
Sentinel-5P	TROPOspheric monitoring instrument (TROPOMI)	Band 1—Ultraviolet	270–320	7 × 28
		Band 2—Ultraviolet		7 × 3.5
		Band 3—Visible	320–495	
		Band 4—Visible		
		Band 5—NIR	675–775	
		Band 6—NIR		
		Band 7—SWIR	2305–2385	7 × 7
		Band 8—SWIR		

## Results and discussion

### Land use/land cover detection

Two primary land use/land cover (LULC) classes were identified in the study: cultivated land and urban areas. The cultivated land class comprises all agricultural zones within the study area, including long-established farmlands, actively cultivated fields, and recently harvested fields. The urban class, as delineated in the LULC maps, encompasses residential and industrial developments, including villages, towns, cities, factories, and both newly constructed and pre-existing buildings and infrastructure.

**Fig. 3:**
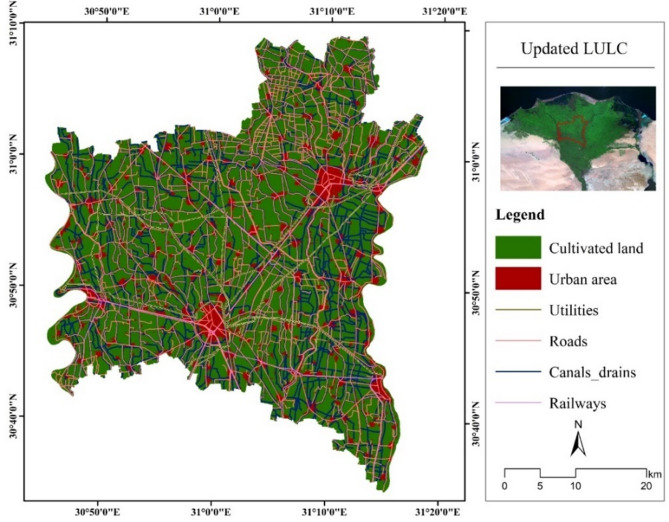
Updated LULC of the study area. (Generated by the authors using Landsat-8 data via ArcGIS version 10.4.1^21^.

Gharbia Governorate is located in the center of the Nile Delta region, characterized by its fertile and productive lands. The agricultural land occupies most of the study area; it exhibits 85.82% (1667.76 km^2^) as shown in Fig. 3; Table 3. The urbanized areas occupy 275.51 km^2^ (14.18%) and are embedded in the agricultural area, connected by a dense road network. The study area is covered by irrigation and drainage canal networks, which are well distributed in the agricultural area. The Nile River is the irrigation source in this region.

**Table 3:** Area of LULC classes.

Land cover	Area (km^2^)	Percentage (%)
Cultivated land	1667.76	85.8
Urban area	275.51	14.2

### Monitoring land use/land cover changes

**Fig. 4:**
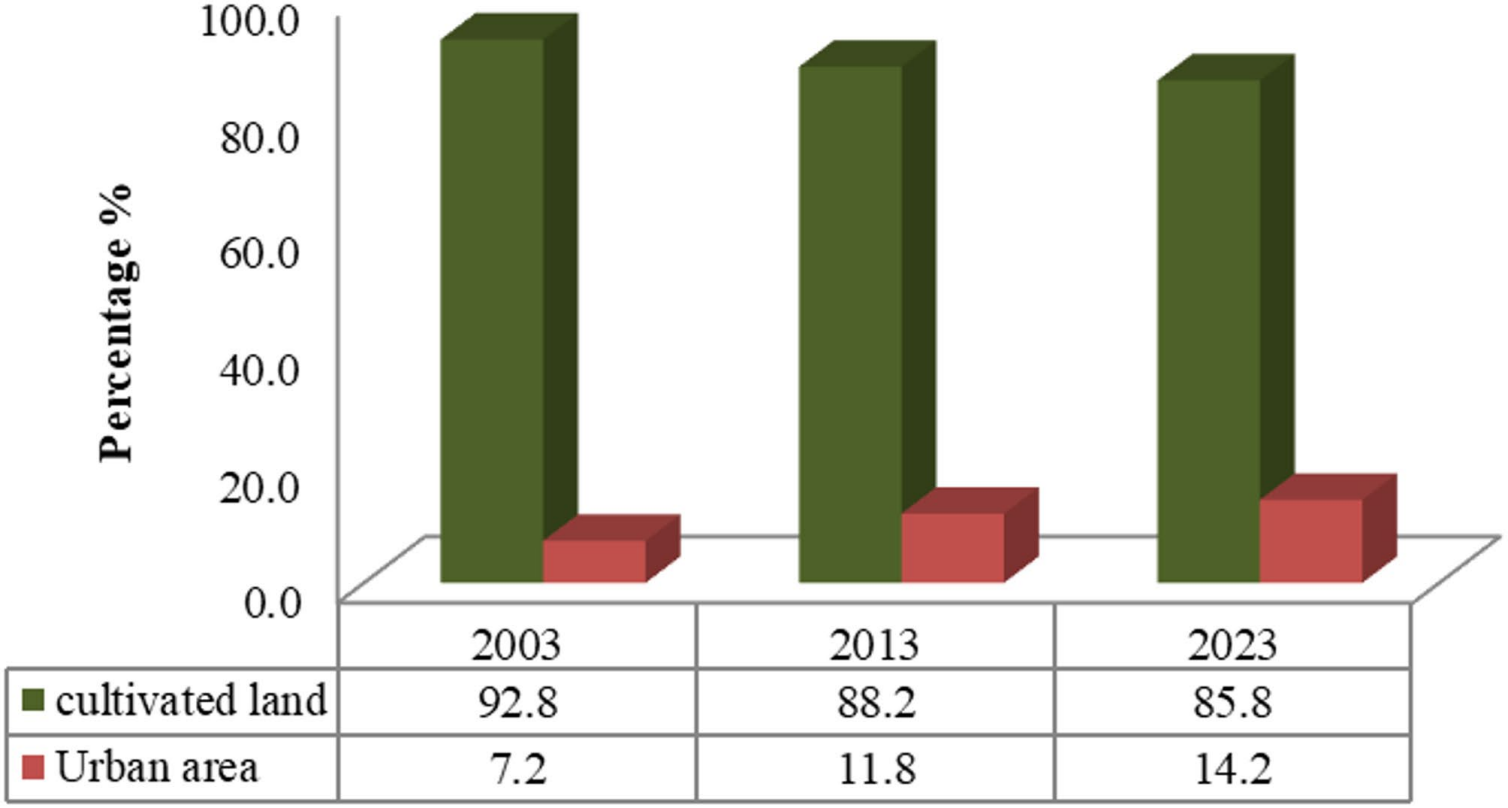
Percentage of LULC for years 2003, 2013, and 2023.

**Fig. 5:**
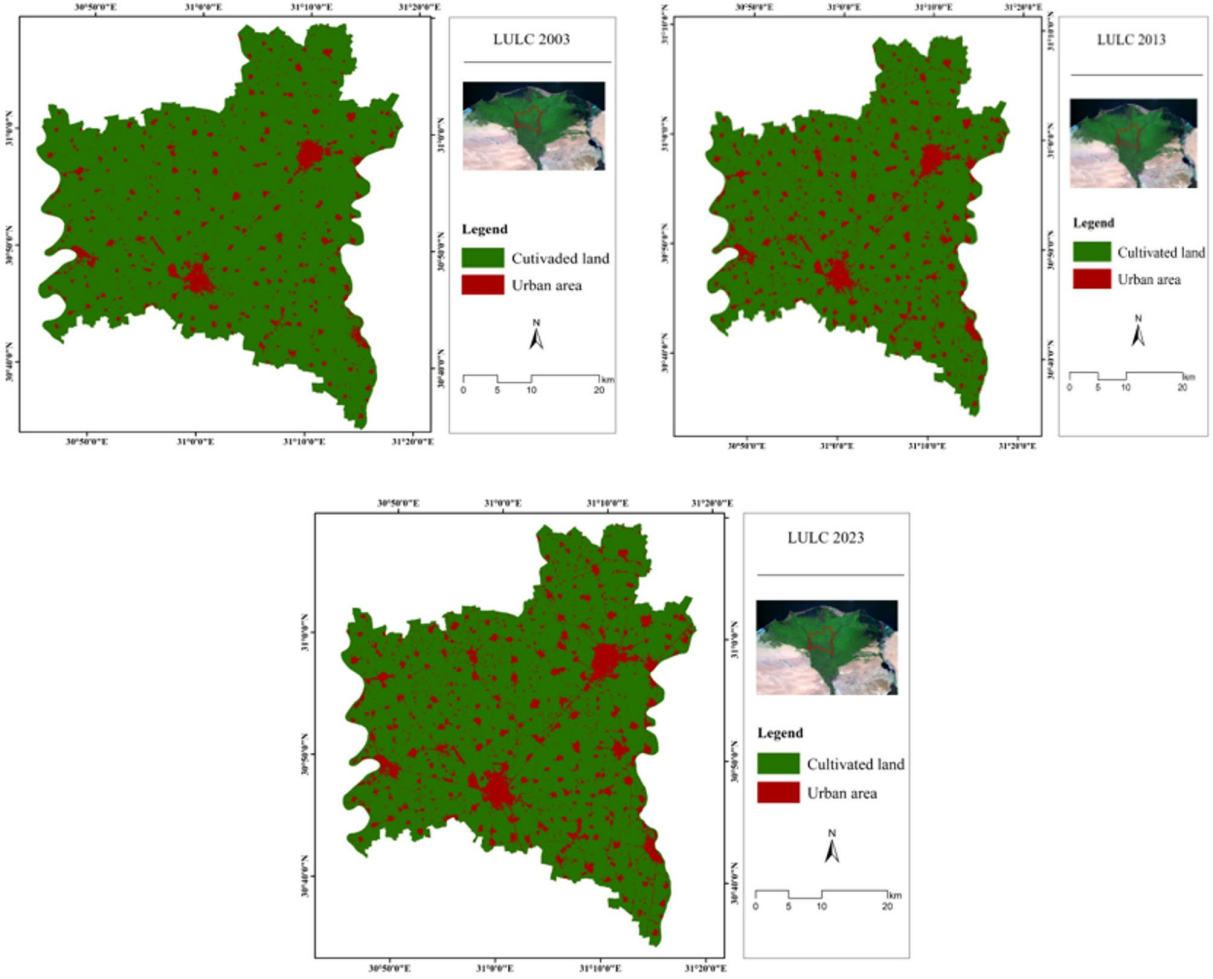
LULC map for the years 2003,2013, and 2023. (Generated by the authors using Landsat-5&8 data via ArcGIS version 10.4.1^21^.

One of the key applications of remotely sensed data is the detection of LULC changes, which plays a vital role in monitoring environmental transformations and assessing the impacts of human activities on land use. Although modernization and urban expansion are central to development planning, their adverse environmental consequences are often underemphasized. This study analyzed LULC changes over two distinct periods, 2003–2013 and 2013–2023, to capture long-term trends and dynamics. Significant temporal variations in land cover were observed across the 20-year study period. The extent of reduction or gain in each LULC class was quantified to determine the rate of change, as illustrated in Table 4; Figs. 4 and 5. These findings offer critical insights into both the spatial and temporal patterns of land cover transformation in the study area.

The results revealed a consistent expansion of urban areas over both study periods. During the first period (2003–2013), urban land increased significantly from 139.57 to 230.07 km^2^, primarily at the expense of cultivated land, which declined from 1803.70 to 1713.20 km^2^. In the second period (2013–2023), urban expansion continued, reaching 275.51 km^2^, while cultivated land further declined to 1667.76 km^2^. This corresponds to a reduction in cultivated area by 4.66% during the first period and 2.34% during the second, as presented in Table 5. These findings suggest a more pronounced agricultural land loss in the first decade compared to the second. Overall, the study indicates that Gharbia Governorate experienced substantial urban growth over the 20-year timeframe. Comparable findings were reported by Mostafa et al.^31^, who observed a notable 147 km^2^ increase in built-up land over a 27-year period.

**Table 4:** The areas of LULC for the years 2003, 2013, and 2023.

Class	2003		2013		2023	
	km^2^	%	km^2^	%	km^2^	%
Cultivated land	1803.7	92.8	1713.2	88.2	1667.76	85.82
Urban area	139.3	7.2	230.1	11.8	275.5	14.18

**Table 5:** Annual change (%) in LULC classes during the periods of study (2003–2023).

Class	2003–2013 (%)	2013–2023 (%)
Cultivated land	− 4.66	− 2.34
Urban area	4.66	2.34

### Air parameter assessment

Using the combined MODIS AOD dataset, daily PM_2.5_ data for 2023 were derived. Results indicated that the PM_2.5_ mean varied from 7.12 to 8.66 µg/m^3^ during the study period. Table 6 shows that the average PM_2.5_ (µg/m^3^) across seasons follows this order: autumn (8.66 µg/m^3^) > spring (8.53 µg/m^3^) > winter (7.77 µg/m^3^) > summer (7.12 µg/m^3^). This variation may be attributed to seasonal differences in aerosol composition and sources, as suggested by Handschuh et al.^32^. Notably, all observed PM_2.5_ levels remained significantly below the Egyptian daily permissible limit of 100 µg/m^3^, as stipulated in Egyptian Gazette No. 199, issued on August 28, 2011.

**Fig. 6:**
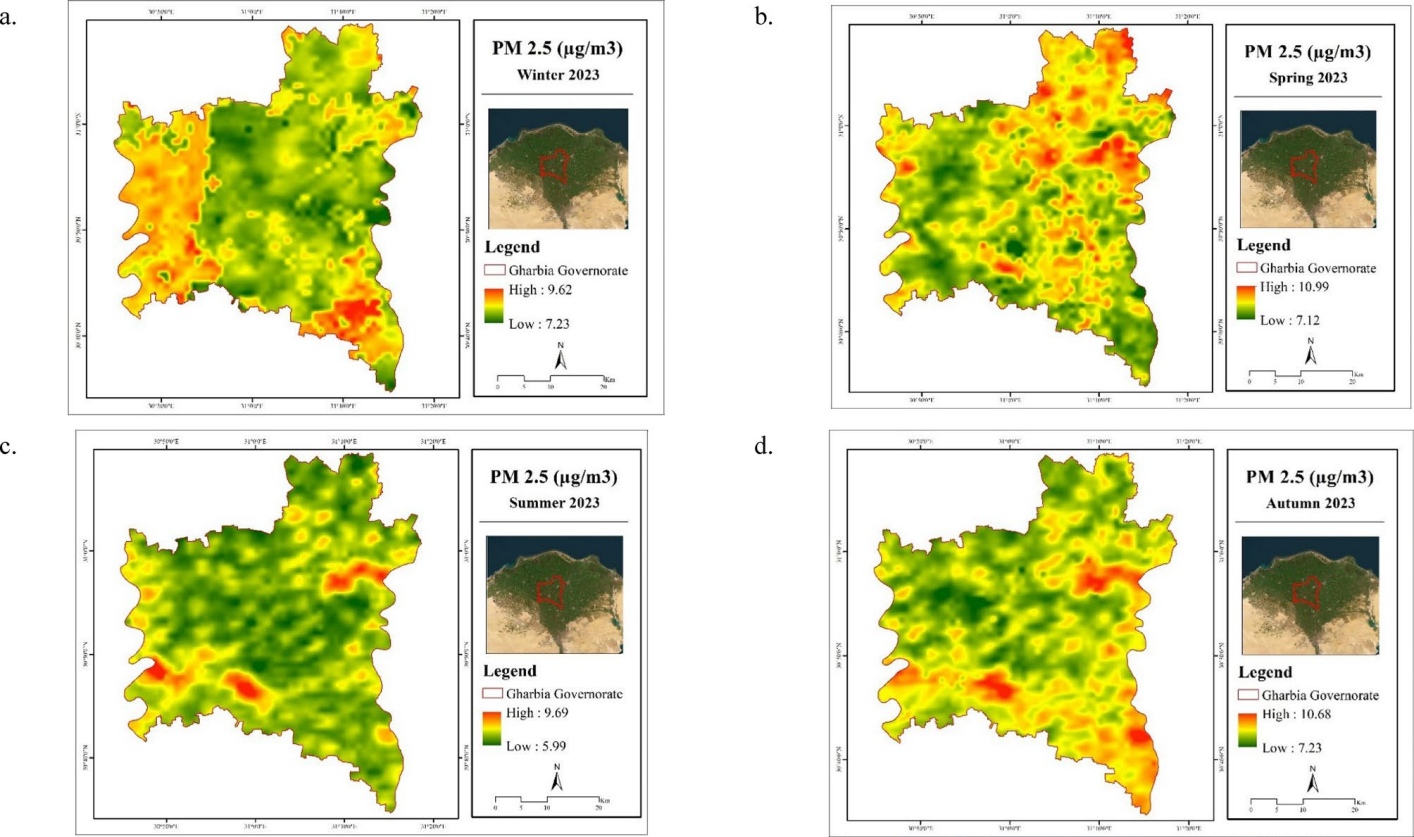
Spatial distribution maps for seasonal mean of PM_2.5_ in Gharbia Governorate during winter (**a**), spring (**b**), summer (**c**), and autumn (**d**) 2023. (Generated by the authors using MODIS data via ArcGIS version 10.4.1^21^.

The spatial distribution of PM_2.5_ concentrations across Gharbia Governorate is illustrated in Fig. 6. The PM_2.5_ maps reveal a consistent seasonal pattern during autumn, winter, and summer, with elevated concentrations primarily concentrated in the western and southeastern regions. Conversely, in the spring season, the highest PM_2.5_ levels were observed in the northeastern part of the governorate. As expected, densely populated urban areas exhibited notably higher PM_2.5_ concentrations compared to rural regions, which generally recorded lower levels of fine particulate matter.

These spatial patterns are consistent with the findings of Yang and Jiang^33^, who investigated the relationship between LULC types and PM_2.5_ concentrations. Their study demonstrated that built-up areas are typically associated with higher PM_2.5_ levels, reinforcing the current observation that urbanization plays a significant role in increasing particulate matter pollution.

**Table 6:** Statistics of PM_2.5_ (µg/m^3^) in Gharbia Governorate during 2023.

Season	PM_2.5_ (µg/m^3^)		
	Min	Max	Mean
Winter	7.23	9.62	7.77
Spring	7.12	10.99	8.53
Summer	5.99	9.69	7.12
Autumn	7.23	10.68	8.66

**Fig. 7:**
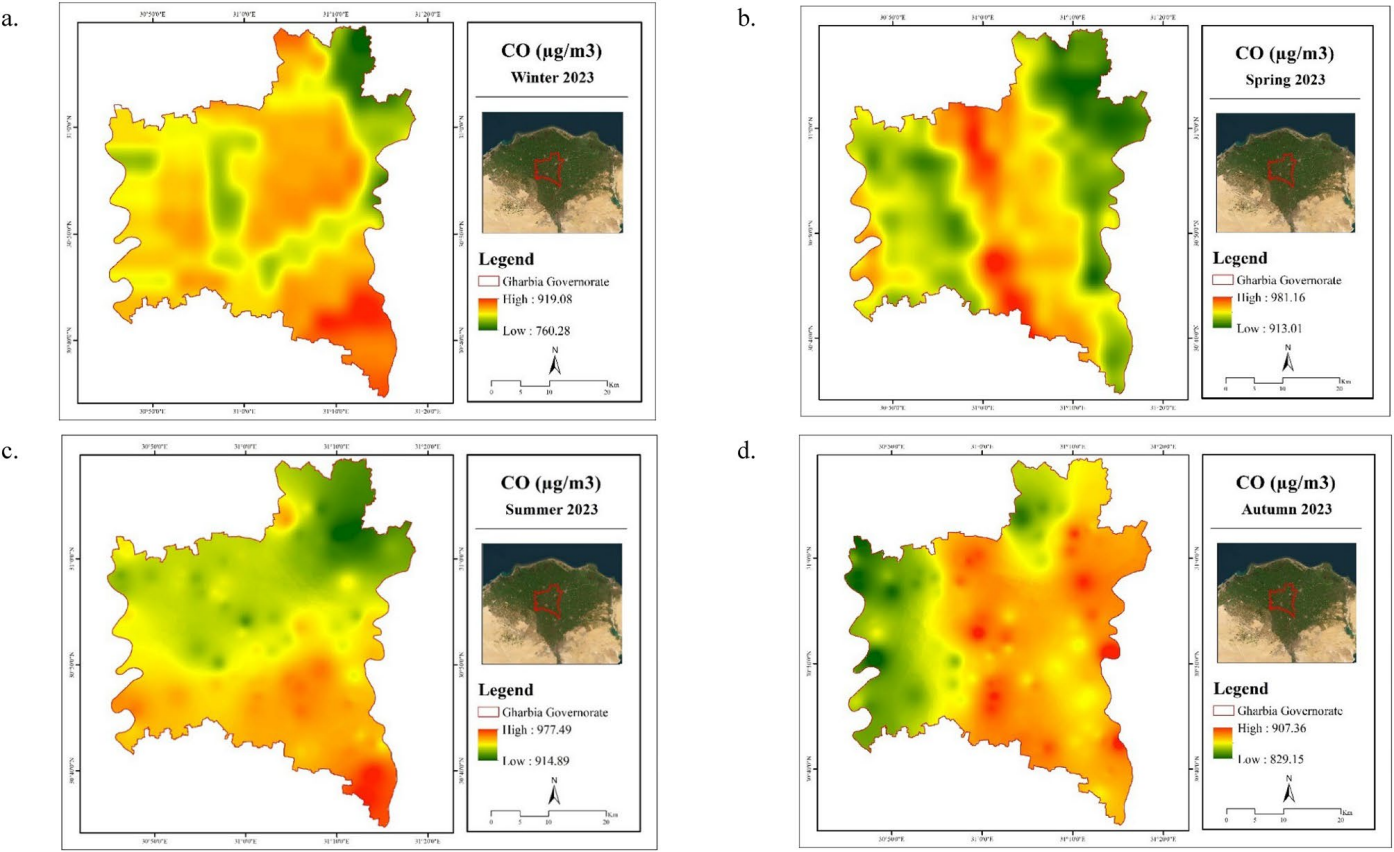
Spatial distribution maps for seasonal mean of CO in Gharbia Governorate during winter (**a**), spring (**b**), summer (**c**), and autumn (**d**) 2023. (Generated by the authors using Sentinel-5P data via ArcGIS version 10.4.1^21^.

Seasonal average concentrations of tropospheric carbon monoxide (CO) in Gharbia Governorate during 2023 are summarized in Table 7. The highest CO levels were recorded in the summer, reaching 946.79 µg/m^3^, while the lowest concentrations were observed during the winter season at 855.70 µg/m^3^. The spatial distribution of tropospheric CO across the governorate is depicted in Fig. 7. Visual analysis reveals that certain areas experienced significantly elevated CO concentrations, with the majority of the governorate exhibiting relatively high values. Notably, the southern regions showed a pronounced accumulation of CO during the summer months.

The elevated atmospheric CO levels observed in summer, compared to winter, may be attributed to a combination of environmental and anthropogenic factors. One contributing factor is the increased rate of photosynthetic activity during spring and summer, which lowers CO₂ concentrations and may influence CO dynamics in the atmosphere^34^. Furthermore, the relatively unstable atmospheric conditions typical of summer promote vertical mixing and pollutant dispersion, allowing CO levels to rise due to ongoing emissions. In contrast, winter’s stable atmospheric conditions can hinder vertical air movement, leading to pollutant accumulation near the surface^35^. Seasonal variations in meteorological parameters and emission sources, such as vehicular activity, industrial processes, and biomass burning, also play a significant role in determining the observed CO concentration patterns^36^.

**Table 7:** Statistics of CO (µg/m^3^) in Gharbia Governorate during 2023.

Season	CO (µg/m^3^)		
	Min	Max	Mean
Winter	760.28	919.08	855.70
Spring	913.01	981.16	942.99
Summer	914.89	977.49	946.79
Autumn	829.15	907.36	870.25

Table 8 shows the seasonal average of tropospheric NO_2_ during the study period. Non-noticeable variation was found between the average of all seasons. However, autumn has experienced relatively higher concentrations, attaining 3.13 µg/m^3^. Autumn season is the time of burning rice straw since there is a greater chance of inversion conditions, and the air stream is not dispersing emissions properly. Methane and NO_2_ are two of the major greenhouse gases released during the field burning of rice straw^37^.

**Fig. 8:**
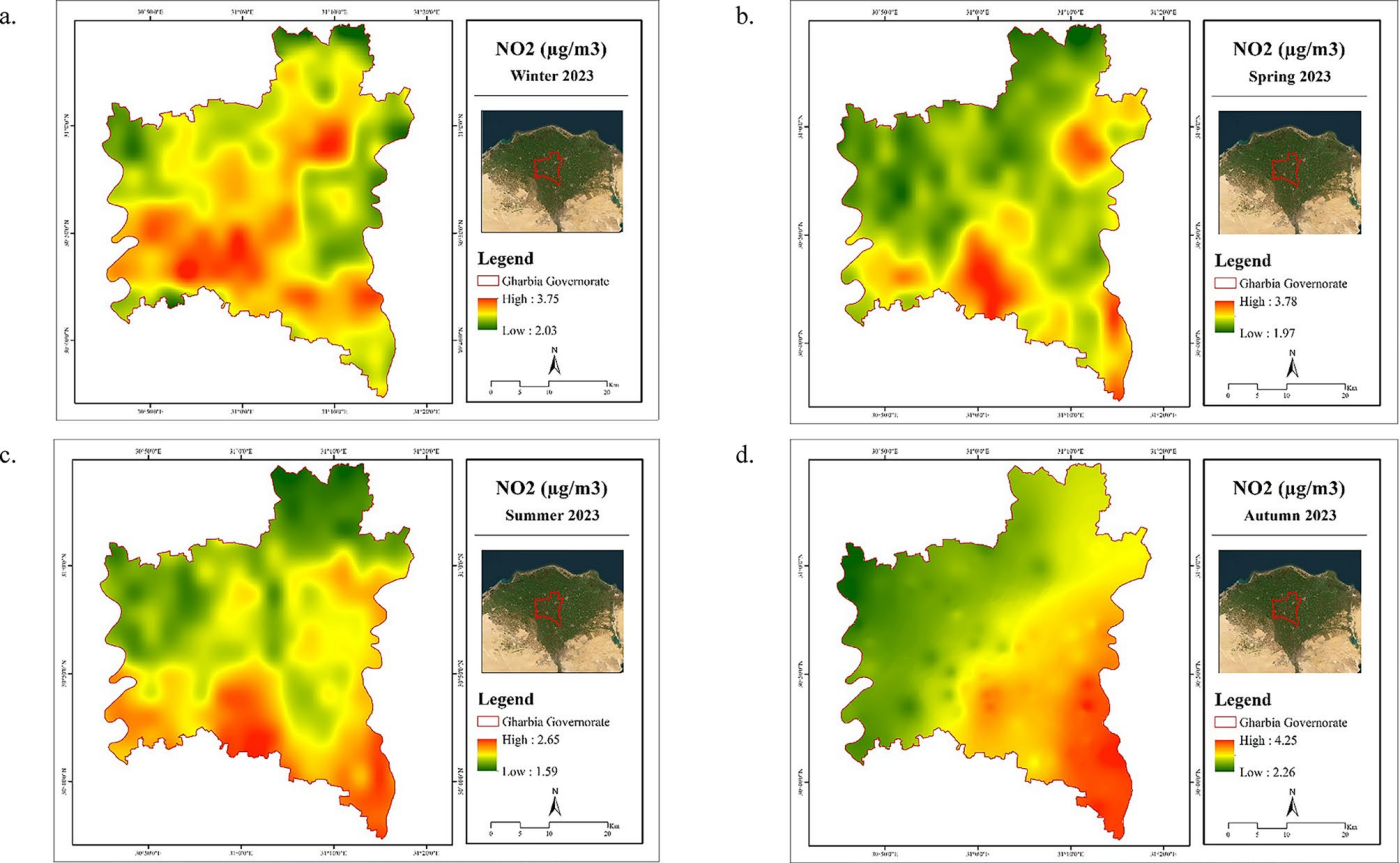
Spatial distribution maps for seasonal mean of NO_2_ in Gharbia Governorate during winter (**a**), spring (**b**), summer (**c**), and autumn (**d**) 2023. (Generated by the authors using Sentinel-5P data via ArcGIS version 10.4.1^21^.

Figure 8 displays the spatial distribution of seasonal averages of tropospheric NO_2_ over Gharbia Governorate in 2023. It was observed that the governorate experienced widespread elevated NO_2_ levels, particularly in the southern region during winter. Additionally, the southern parts of the governorate continued to record the highest NO_2_ levels in other seasons. The expansion of urban areas into agricultural lands, due to the lack of desert space in Gharbia Governorate for urban growth, contributes to the release of pollutants, including NO_2_, into the atmosphere^38^.

Mostafa et al.^39^ found that there is a rapid urbanization trend in Gharbia Governorate, leading to a diminution of agricultural land and an increase in built-up areas. This urban sprawl negatively impacts environmental sustainability and may contribute to higher levels of NO_2_ in the region. Additionally, industrial activities and transportation emissions in urban areas can also contribute to higher levels of NO_2_.

**Table 8:** Statistics of NO_2_ (µg/m^3^) in Gharbia Governorate during 2023.

Season	NO_2_ (µg/m^3^)		
	Min	Max	Mean
Winter	2.03	3.75	2.97
Spring	1.97	3.78	2.83
Summer	1.59	2.65	2.08
Autumn	2.26	4.25	3.13

**Fig. 9:**
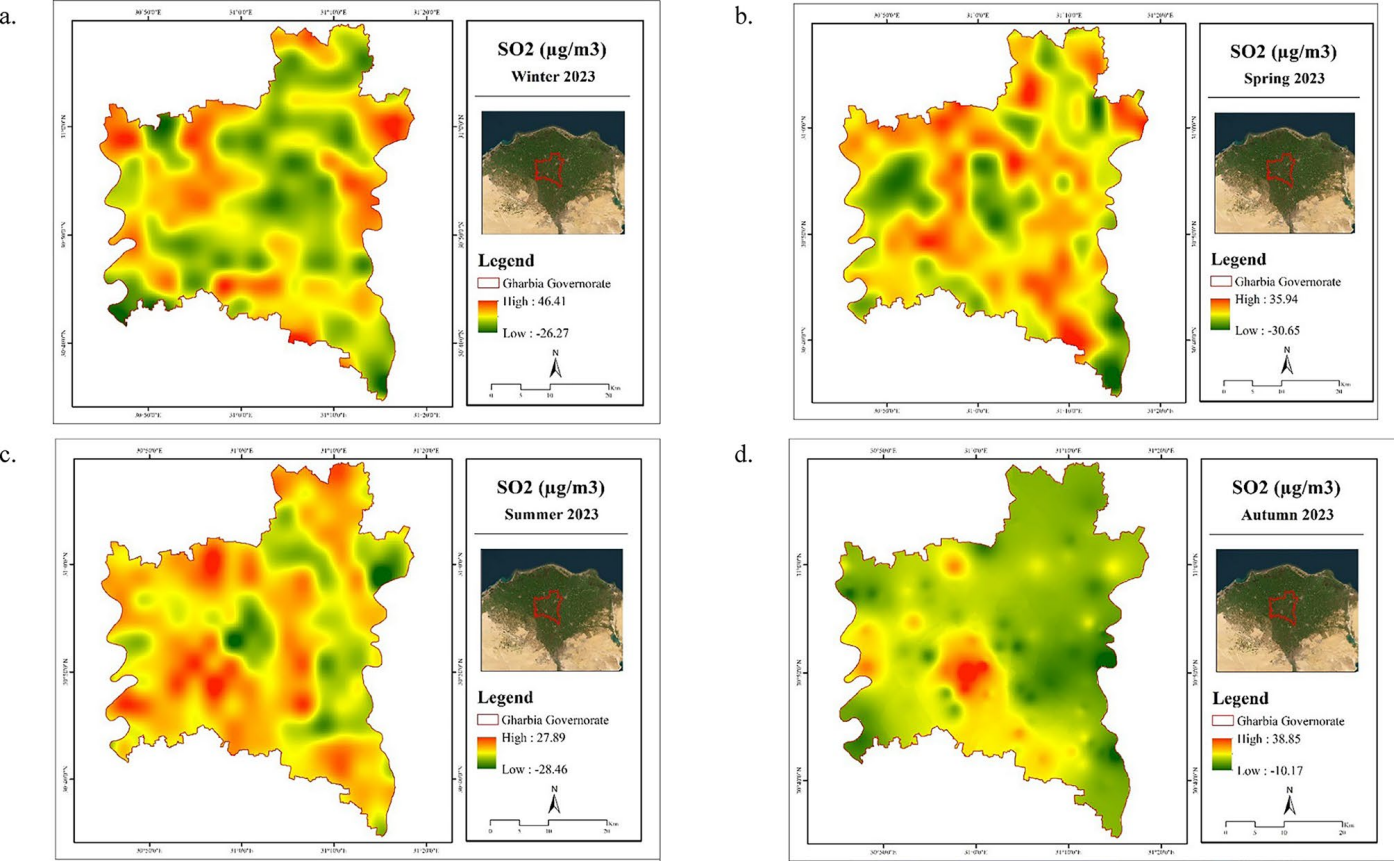
Spatial distribution maps for seasonal mean of SO_2_ in Gharbia Governorate during winter (**a**), spring (**b**), summer (**c**), and autumn (**d**) 2023. (Generated by the authors using Sentinel-5P data via ArcGIS version 10.4.1^21^.

A slight increase in seasonal SO_2_ concentrations was observed, with values ranging from 5.56 to 8 µg/m^3^, and the highest seasonal mean was recorded during winter (8 µg/m^3^), as shown in Table 9. The elevated SO_2_ levels in winter may be attributed to intensified use of heating systems that rely on solid fuels^40^. As illustrated in Fig. 9, high concentrations of tropospheric SO_2_ were distributed widely across Gharbia Governorate during spring, summer, and winter, with notably elevated values concentrated in the central part of the region. These increased SO_2_ levels are primarily driven by the combustion of fossil fuels, like coal, oil, and natural gas, which are widely recognized as the dominant sources of anthropogenic SO_2_ emissions^41^. Such activities can result in hazardous pollution levels, particularly near industrial zones and power plants. Although the study by Abbas and Rajab^42^ focused on the spatial and temporal distribution of anthropogenic SO_2_ emissions in Iraq, the findings offer relevant insights and can be cautiously extrapolated to explain similar emission patterns observed in Gharbia Governorate. Collectively, the results suggest that fossil fuel combustion plays a significant role in the elevated SO_2_ concentrations recorded in the study area.

**Table 9:** Statistics of SO_2_ (µg/m^3^) in Gharbia Governorate during 2023.

Season	SO_2_ (µg/m^3^)		
	Min	Max	Mean
Winter	− 26.27	46.41	8
Spring	− 30.65	35.94	7.83
Summer	− 28.46	27.89	5.56
Autumn	− 10.17	38.85	6.17

Generally, the spatial distribution pattern of the examined air pollutants demonstrated that the region was almost synchronized with Kafr El-Zayat, Tanta, and El-Mahalla El-Kubra, characterized by elevated concentrations of air pollutants. This may be due to the presence of uncontrolled industrial activities (e.g., fertilizers, chemicals, and pesticides), which have recently caused several resident complaints in Kafr El-Zayat. Furthermore, in the majority of agricultural areas throughout the governorate, such as Tanta and El-Mahalla El-Kubra, burning rice straw as a method of disposal of large amounts of harvested rice results in a considerable amount of air pollutants (i.e., PM_2.5_).

According to CAPMAS report (2020), Gharbia Governorate produced approximately 341.9 thousand tons of rice, accounting for 9.1% of Egypt’s total rice production. The cultivated area dedicated to rice in the governorate was 99.6 thousand feddans, representing 7.9% of the national total. Notably, around 43.9 thousand tons of rice straw, equivalent to 2.1% of the total straw generated, were subjected to open-field burning. Numerous studies have documented the adverse environmental impacts of rice straw burning, highlighting its significant contribution to air pollution through the release of harmful pollutants. This practice is known to deteriorate ambient air quality, disrupt climate systems, and pose serious risks to public health^43–45^. Nevertheless, it is important to note that the observed concentrations of PM_2.5_, CO, NO_2_, and SO_2_ in this study remained below the threshold limits established by the World Health Organization (WHO, 2023) for ambient air quality (www.eea.europa.eu).

### Urban sprawl effects on air quality

**Fig. 10:**
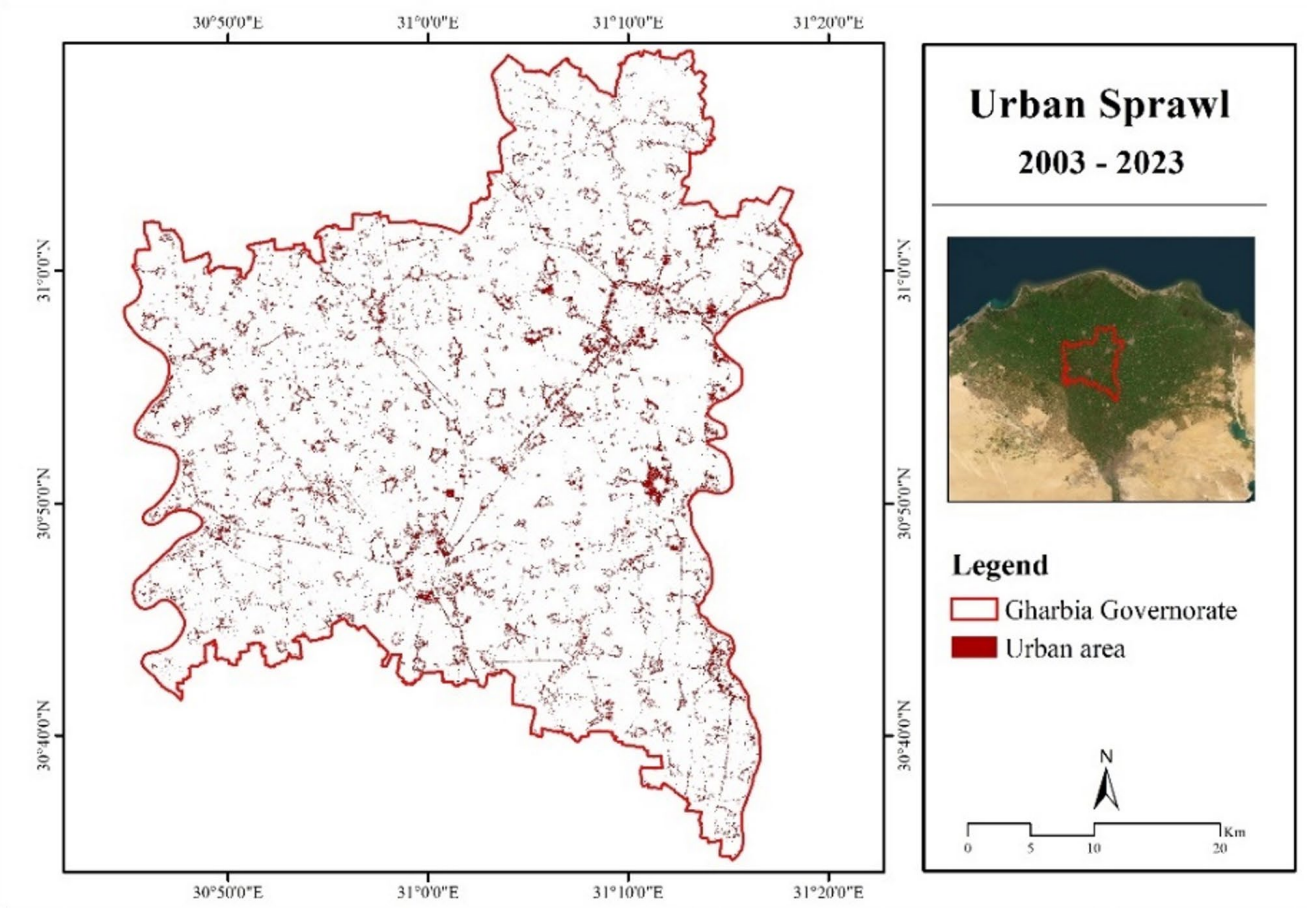
Urban sprawl across Gharbia Governorate from 2003 to 2023. (Generated by the authors using Landsat 5 & 8 data via ArcGIS version 10.4.1^21^.

Figure 10 illustrates urban sprawl from 2003 to 2023, covering a total area of 134.74 km^2^. The examined air pollutants were specifically measured in these areas and are shown in Table 10. The average values of the investigated air quality parameters in the expanded areas, resulting from urban development and sprawl, clearly increased. This suggests that urban sprawl is associated with higher concentrations of air pollutants due to urban growth, human activities, and increased emissions. Urbanization alters meteorological conditions, resulting in changes in the levels of air pollutants, including PM_2.5_, CO, and NO_2_. Urban expansion also raises air temperature and the ventilation index, which influences how pollutants spread^46, 47^.

Several studies in European Union cities, China, and Indonesia reveal that urban sprawl contributes to increased air pollution levels. Factors such as built-up area development, population density, and residential discontinuity play significant roles in exacerbating pollutant concentrations^48–50^. Additionally, research also indicates that air pollutant concentrations are positively correlated with increasing built-up areas and negatively correlated with forests, suggesting that urban sprawl plays a significant role in air quality deterioration^51,52^. Similarly, Nagy et al.^30^ accomplished a study on another Egyptian Delta governorate and noted that the deteriorating regions of air quality were related to the regions with anthropogenic contributions.

**Table 10:** Mean of air pollutants over the area of urban sprawl across Gharbia Governorate.

Season	Air pollutants (µg/m^3^)			
	PM_2.5_	CO	NO_2_	SO_2_
Winter	8.17	856.40	3.01	8.19
Spring	8.73	943.60	2.91	7.84
Summer	7.30	946.68	2.12	5.13
Autumn	8.84	872.49	3.19	6.25

## Conclusion

To support environmental sustainability in Gharbia Governorate, this study aims to monitor land use/land cover (LULC) changes over the past two decades and assess their impact on air quality. Multiple satellite datasets were utilized to assess changes in both land use and air quality in Gharbia Governorate. A Maximum Likelihood Classifier (MLC) was applied to perform supervised classification of remotely sensed data for detecting LULC changes. In parallel, data from Aqua, Terra, and Sentinel-5P satellites were used to estimate atmospheric concentrations of PM_2.5_, CO, NO_2_, and SO_2_. The LULC classification results indicated that agricultural land remains the dominant land cover type across the governorate. The urban areas increased from 139.57 to 230.07 km^2^ in the first duration and continued to increase, reaching 275.51 km^2^ in the second duration. Results of air parameters showed that PM_2.5_ and NO_2_ recorded a relatively high level in autumn, attaining 8.66 and 3.13 µg/m^3^, respectively. However, elevated levels of CO and SO_2_ were found in summer (946.79 µg/m^3^) and winter (8 µg/m^3^), respectively. The findings clearly show that urban sprawl is a major contributor to deteriorating air quality, particularly in the newly developed urban areas. Combined with natural and anthropogenic emissions, such as desert dust and agricultural burning, this urban expansion intensifies the environmental pressures on the region. In conclusion, urban growth in Gharbia Governorate has had a measurable and negative impact on both land resources and air quality. The use of space-borne technologies in this study has proven effective in capturing the long-term effects of land use change on environmental conditions. These insights are critical for guiding future planning and sustainable development strategies. One key limitation of this study is the lack of long-term air quality data spanning the full 2003–2023 period. Due to the limited availability of high-resolution satellite-based pollutant data prior to 2017, the air quality analysis is restricted to 2023. As a result, the study focuses on the spatial correlation between recent land use changes and pollution levels, rather than a temporal trend analysis. Future research should aim to incorporate multi-temporal air quality datasets, where available, to strengthen the assessment of long-term urbanization impacts.

## Data Availability

Availability of data and materials: All data generated or analyzed during this study are included and available in this article.
